# Nasopharyngeal cancer risk assessment by country or region worldwide from 1990 to 2019

**DOI:** 10.1186/s12889-024-19228-9

**Published:** 2024-07-18

**Authors:** Xian Wei, Biaoyou Chen, Zihao Wang, Peng Zhao, Xuwei Duan

**Affiliations:** Department of Head and Neck Surgery, Guangxi Medical University Cancer Hospital, 71 Hedi Road, Nanning, 530021 China

**Keywords:** Nasopharyngeal carcinoma, Global burden of disease (GBD), Burden of diseases, EAPC

## Abstract

**Background:**

Nasopharyngeal carcinoma (NPC) is 22nd most common cancer that occurs all over the world, but the prevalence rate can exhibit significant geographical differences. The Global Burden of Disease (GBD) database provides data related to the incidence, mortality, and disease burden of NPC worldwide from 1990 to 2019. We have designed this study in order to evaluate the potential effectiveness of health care policies and strategies for NPC prevention, diagnosis and treatment in different countries or regions around the world.

**Methods:**

We used for the first time two distinct indicators, EAPC-ASIR and EACP-ASDR, to perform cluster analysis on 200 countries or regions around the world.

**Results:**

200 countries or regions could be divided into five diverse groups. Group 1: The incidence rate showed an increasing trend whereas the mortality rate depicted a decreasing trend. Group 2: Morbidity as well as mortality showed a slight increase; Group 3: Morbidity as well as mortality increased significantly; Group 4: Morbidity and mortality decreased significantly; Group 5: Both morbidity as well as mortality decreased slightly. Moreover, in the context of a global decline in NPC incidence, mortality and disease burden, Group 3 countries, including: “Turkmenistan”, “Bosnia and Herzegovina”, “Dominican Republic”, “Bulgaria”, “Lesotho”, “Cabo Verde”, “Romania”, “Cuba”, “Jamaica”, “Azerbaijan”, “Uzbekistan”, “Chad”, “Belize” and “Ukraine” displayed a significant increase in morbidity, mortality, and disease burden, thus indicating a dangerous trend.

**Conclusion:**

It is suggested that the medical and health policies formulated by the countries in Group 3 for NPC, as well as their capacity for conducting censuses, preventing, diagnosing, and treating diseases, need to be substantially strengthened.

## Introduction

Nasopharyngeal carcinoma (NPC) is a malignant tumor that has been found to occur worldwide, but with significant geographical differences in incidence [1], and is closely related to the EBV virus [2]. The anatomical location of NPC is rather deep, and early symptoms are mild as well as atypical, leading to a high misdiagnosis rate during the early stages of NPC. Therefore, most cases are already identified in the middle and advanced stages of cancer when successfully clinical diagnosed [3]. It has been reported that NPC is most commonly diagnosed in the locally advanced stages, with lymph node metastases occurring in up to 90% of patients whereas approximately 5–10% of patients present with the distant metastases [4], and if the cancer metastasizes to vital tissues and organs, the prognosis is very poor [5]. Radiation therapy is the main treatment modality currently being used for the management of NPC patients [6], and intensity-modulated radiation therapy has yielded improved outcomes [7]. However, due to the proximity of important organs to the radiation field, patients with NPC often experience several serious complications such as anosmia, diminution of vision, decreased hearing [8], and trismus [9] after the systemic treatment with radiation and chemotherapy. These complications often exhibit a high incidence rate and can occur early or at distinct stages of the treatment, thereby not only reducing the quality of life for patients but also imposing a heavy burden on the society. The prognosis for localized NPC patients is promising, with a 5-year Overall survival (OS) of 93.2% and an 8-year OS of 85.5% [10]. However, due to the lack of suitable tumor biomarkers that can be used clinically and the non-specific clinical symptoms associated with NPC, early-stage carcinoma is often easy to miss the diagnosis. Thus, thorough understanding the global and regional epidemiological characteristics of NPC and improving its awareness can aid with early diagnosis and treatment of affected patients. For instance, using various screening methods such as detecting EBV antibodies in the serum and nasal endoscopy examination when encountering symptoms similar to NPC in the clinical practice can contribute to significantly improve the survival rate of NPC and reduce the burden on society.

However, so far, no one is currently paying attention to changes at the national level of the NPC. Since the nation is the power center of implementing health care, analyses of the changing trend of NPC morbidity and mortality at the national level can have positive significance for each country or region, and can aid to better formulate the various prevention strategies according to the different national conditions.

The Global Burden of Disease (GBD) Study provides an annually updated resource to study the disease-related morbidity and mortality worldwide. The GBD now provides estimates for each year from 1990 to the present for 371 different diseases and injuries, as well as 3,499 clinical outcomes (sequelae) related to those diseases and injuries, for 204 different countries and territories and for the subnational units in more than 20 countries [11]. The full time series produced in each round of the GBD is updated on an annual basis [12]. In this study, we have elegantly analyzed the detailed statistical data on NPC from 1990 to 2019, aiming to reveal the global geographical distribution and 30-year trends of NPC, which can help the decision-makers assess the disease burden and allocate limited medical resources effectively.

## Materials and methods

GBD database is a collaborative effort that involves the collection and integration of the global disease and population health-related data from the diverse sources. The data including eight data indicators: Estimate (cause of death or injury), measure (deaths, incidence, prevalence, years lived with disability (YLDs), years of life lost (YLLs) and years lived with disability (YLDs), maternal mortality ratio), metric (number, percent, rate), location, age, sex, and year. All the data we needed was downloaded from the Global Health Data Exchange (GHDx) query tool (http://ghdx.healthdata.org/gbd-results-tool), including 204 different countries or territories, sex, age, incidence, deaths, disability-adjusted life years (DALYs). As there were 4 countries or territories (Guam, Palestine, South Sudan, Taiwan) that were controversial internationally, their data was excluded, thus resulting in a total of 200 countries or territories included in the current study.

### Statistical analysis

Annual incidence cases, deaths, DALYs, ASRs and corresponding Estimated Annual Percentage Changes (EAPCs) were used to assess the different trends in the incidence and mortality rate of NPC. DALYs are composed of YLLs and YLDs. One DALY can be regarded as 1 lost year of “healthy life” [13]. ASRs include age-standardized incidence rate (ASIR), age-standardized death rate (ASDR) and age-standardized DALY rate (ASYR). The ASRs included in GHDx is considered as an objective indicator to quantify the trends in cancer incidence. Standardization is required for comparison among the several differently age-structured populations or for a certain population over a period of time with its time-dependent age profiles. The trend of ASRs can be reflected by EAPC value, which is often used to measure the various trends in disease and mortality rates, by using a linear model on the log of the ASRs [14] (ASRs includes ASIR, ASDR and ASYR). According to the following regression model: y = β0 + β1χ + ε, where y represents ln(ASRs) and χ refers to the calendar year, EAPC = 100 ×(exp(β1)-1) and its 95% confidence interval (CI) can be obtained [15]. If the EAPC value and its lower limit of 95% CI are both positive, ASRs is considered to be significantly rising. Conversely, if the EAPC value and its upper limit of 95% CI are both negative, ASRs is regarded as displaying a downward trend. Otherwise, ASR is considered as stable.

### Data visualization

The data analysis was managed by open-source software R (version 4.2.2). The data cleaning was deal with packages including tidyverse. The data visualization was performed by using packages including ggplot2, sf, map, and patchwork and so on. Heat map was used to show the variation trends of ASIR and ASDR from 1990 to 2019. GBD database provided seven distinct measures: Deaths, Incidence, Prevalence, DALY, LYY, LYD and maternal mortality ratio. Maternal mortality ratio was outside the scope of our study, and we discovered that deaths and incidence can summarize the data briefly, because DALY, LYY, and LYD were related to the mortality rate, whereas prevalence are associated with incidence. We used the Estimated Annual Percentage Changes in Age-Standardized Incidence Rate for Both sexes (EAPC-ASIR-BOTH) and Estimated Annual Percentage Changes in Age-Standardized Death Rate for Both sexes (EAPC-ASDR-BOTH) reference indicators to evaluate and analyze the both incidence as well as mortality rates of NPC in 200 countries or regions, and then performed clustering based on the results for these countries or regions. Factoextra package was employed in determining the number of clusters and these clusters were used for the subsequent analysis. The world map was used to display the NPC clusters of 200 different countries or territories visually.

## Results

### 200 countries or regions were clustered into 5 different subgroups

**Fig. 1:**
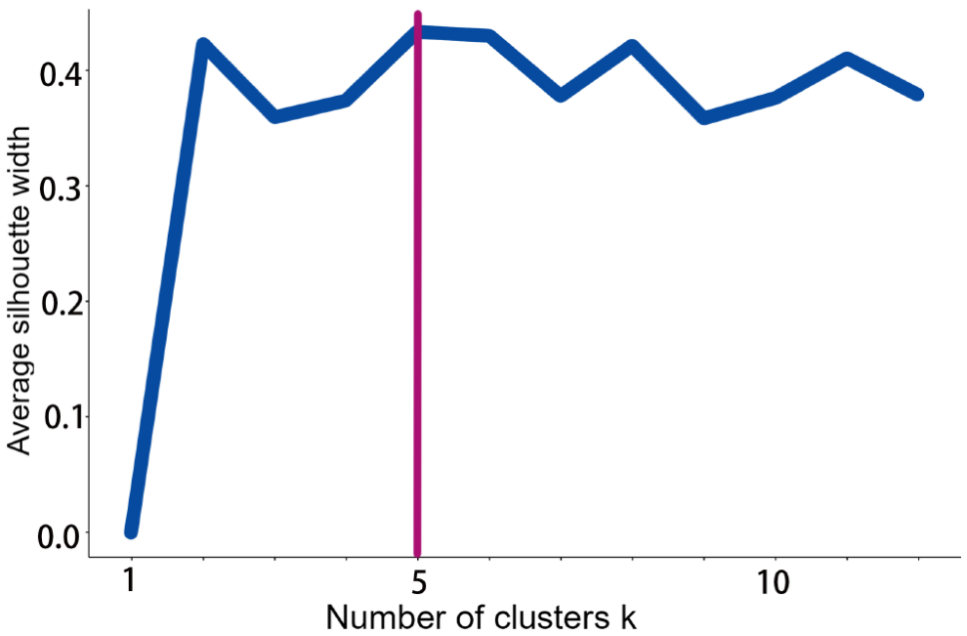
Factoextra package determine the optimal number of clusters, K indicates the optimal number of groups

**Fig. 2:**
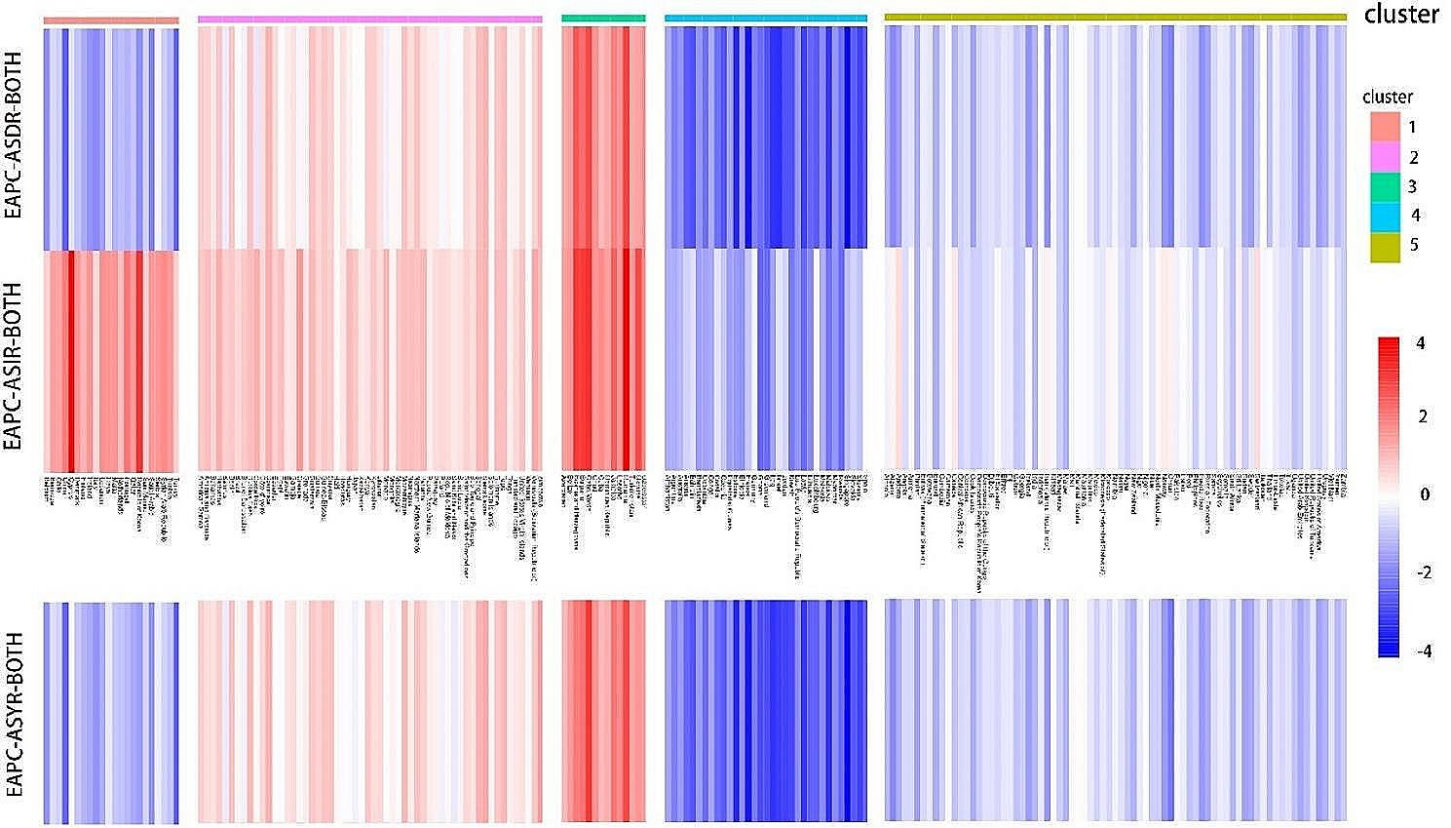
The details of 200 countries or regions divided into 5 groups. EAPC-ASIR-BOTH: Estimated Annual Percentage Changes in Age Standardized Incidence Rate for men and women; EAPC-ASDR-BOTH: Estimated Annual Percentage Changes in Age Standardized Death Rate for men and women; EAPC-ASYR-BOTH: Estimated Annual Percentage Changes in Age Standardized disability-adjusted life Years Rate for men and women

In this study, we used the factoextra package to determine the optimal number of clusters present and applied the K-means method to cluster 200 different countries or regions based on the evaluation criteria of EAPC-ASIR-BOTH and EAPC-ASDR-BOTH. The heatmap revealed that the optimal number of clusters was 5 (Fig. 1), which yielded the most concise grouping information. We then named each subgroup based on their specific characteristics. Group 1, named as “poor control of incidence group,” exhibited an increasing trend in EAPC-ASIR-BOTH and a decreasing trend in EAPC-ASDR-BOTH, thus indicating an increasing incidence rate and a decreasing mortality rate. Group 2, named as the “poor incidence and mortality group,” showed a slight increase in both EAPC-ASIR-BOTH and EAPC-ASDR-BOTH, thus indicating a relatively stable incidence and mortality rate with a slight upward trend. Group 3, named as the “deteriorating incidence and mortality group,” exhibited a significant increase in both EAPC-ASIR-BOTH and EAPC-ASDR-BOTH, thereby indicating a significant increase in both incidence and mortality rates. Group 4, named as the “good incidence and mortality group,” had a significant decrease in both EAPC-ASIR-BOTH and EAPC-ASDR-BOTH, thus suggesting a significant decrease in both incidence and mortality rates. Group 5, named as the “stable incidence and mortality group,” displayed a slight decrease in both EAPC-ASIR-BOTH and EAPC-ASDR-BOTH, thus indicating a relatively stable incidence and mortality rate with a slight downward trend (Fig. 2). The specific list of the countries or regions in each subgroup can be found in the appendix. Additionally, we have also created a heatmap for EAPC-ASYR-BOTH, which demonstrated similar trends to EAPC-ASDR-BOTH. For instance, Group 1 showed a slight downward trend, Group 2 had a slight upward trend, Group 3 exhibited a significant upward trend, Group 4 had a significant downward trend, and Group 5 revealed a slight downward trend (Fig. 2).

### Global geographical distribution of the five groups

**Fig. 3:**
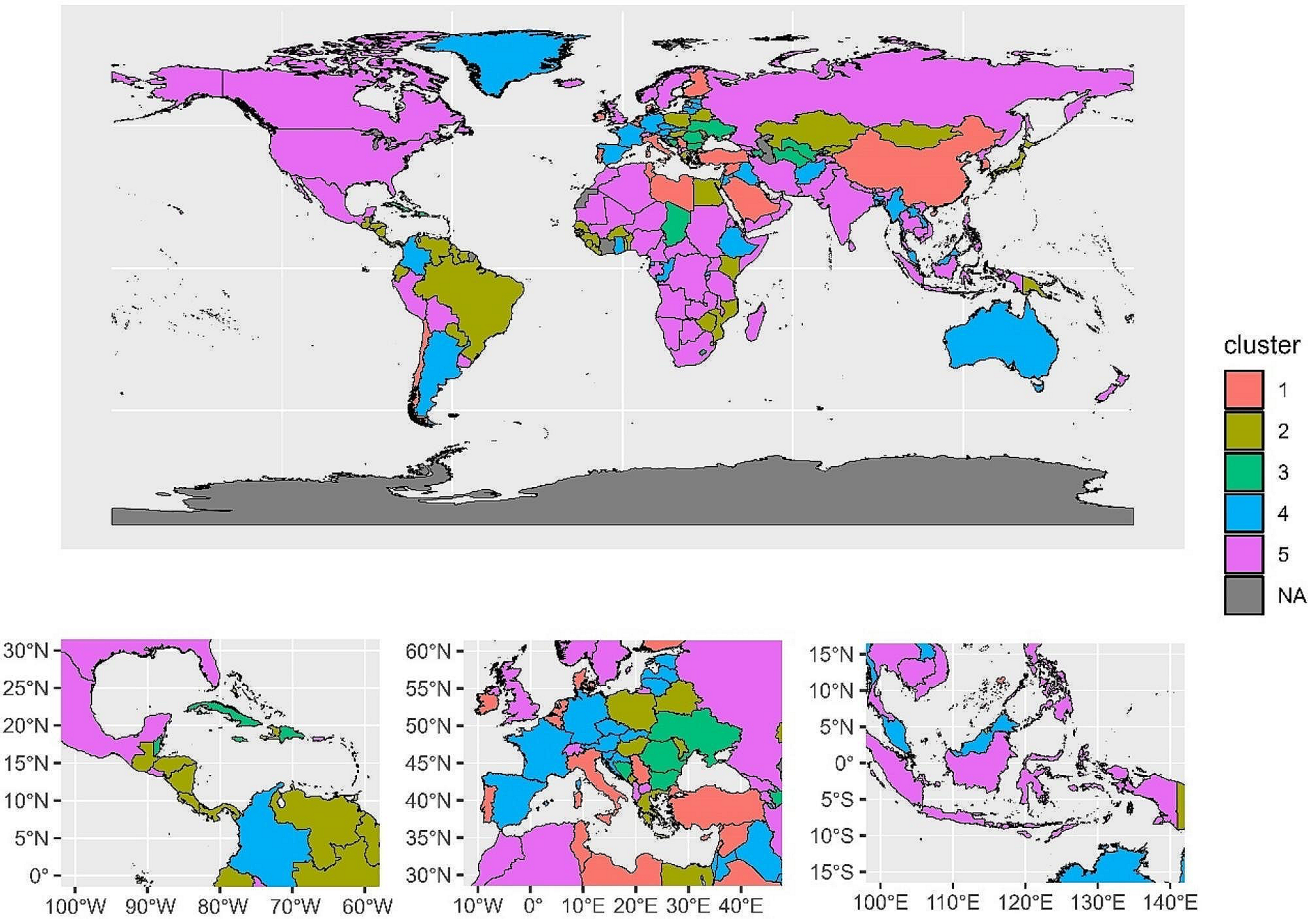
The global distribution of the 5 groups. Different colors represent different groups. NA: Grey areas indicate countries or regions where the GBD is disputed or not included in the statistics

Thereafter, in order to better display the global distribution of the 200 countries or regions included in the five subgroups, we presented the geographical distribution of these five subgroups on a world map. Group 1 included 22 countries or regions, mainly distributed in the countries or regions of Eurasia and Africa between the Tropic of Cancer and 60°N latitude in the Northern Hemisphere. Group 2 consisted of 56 countries or regions, mainly distributed around the equator and near 50°N latitude. Group 3 included 14 countries or regions, Group 4 comprised of 33 countries or regions, and Group 5 includes 75 countries or regions (Fig. 3).

### Alterations in ASIR/ASDR/ASYR for NPC

**Fig. 4:**
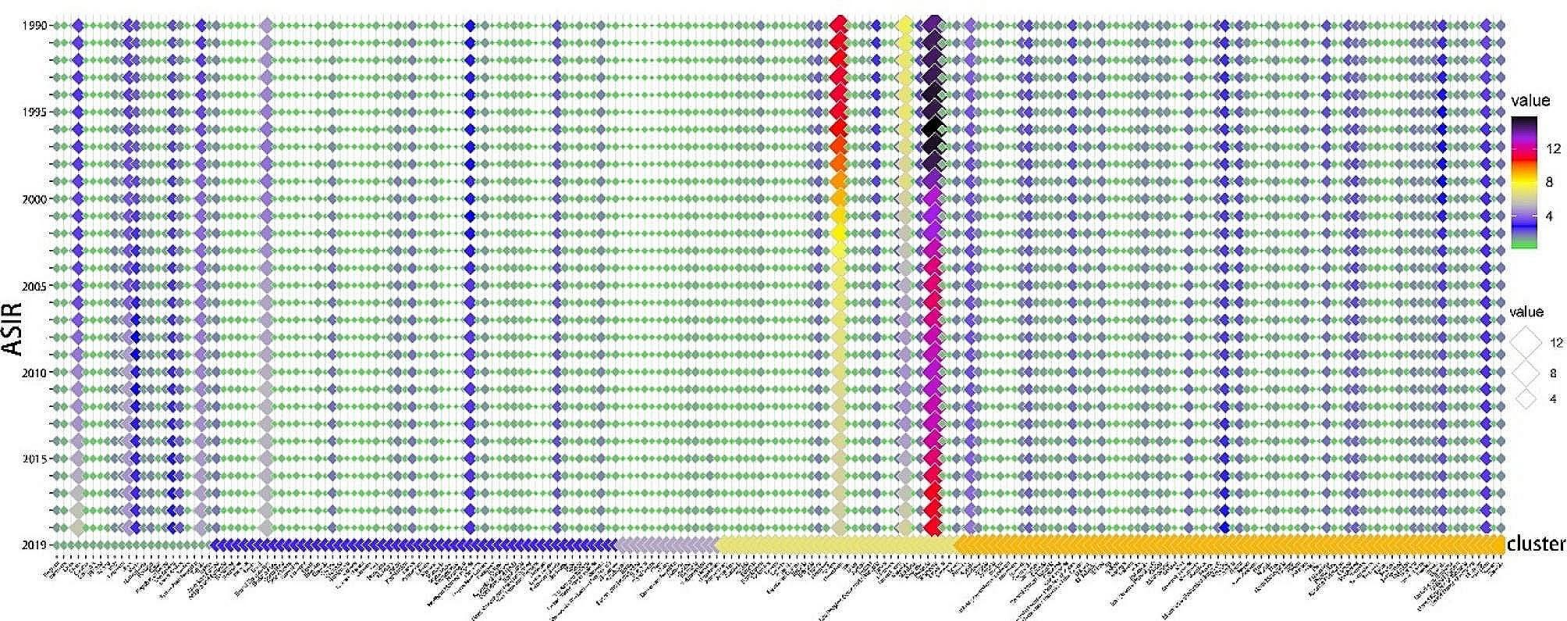
Alterations in ASIR of NPC in 200 countries or regions over the past 30 years

After dividing the 200 countries or regions into five distinct subgroups, we continued to analyze the changes in ASIR, ASDR, and ASYR between 1990 and 2019 for these subgroups and displayed the results by using a bubble chart. Overall, the ASIR in NPC demonstrated an upward trend (global: EAPC-ASIR: 1.13 per 100,000) (Fig. 4). We found that NPC is still a region-specific malignant tumor, with about two-thirds of countries or regions having an ASIR < 1.0 (per 100,000) and maintaining this incidence rate for a considerably long time. Some countries or regions were still able to maintain a high ASIR > 1 (per 100,000). Interestingly, during the 30-year period, it was observed that Singapore, Greenland, Malaysia, Brunei Darussalam, China, Tunisia, Libya, Algeria, Viet Nam and Northern Mariana Islands occupied the top 10 positions in the annual ASIR rankings from 1990 to 2019. In addition, Singapore, Greenland, and Malaysia have long occupied the top three positions in the annual rankings. The most noteworthy is that Singapore (EAPC-ASIR: -1.12 per 100,000) was always ranked first in the rankings every year for 30 years, and its ASIR > 10 (per 100,000), far exceeding other countries or regions.

**Fig. 5:**
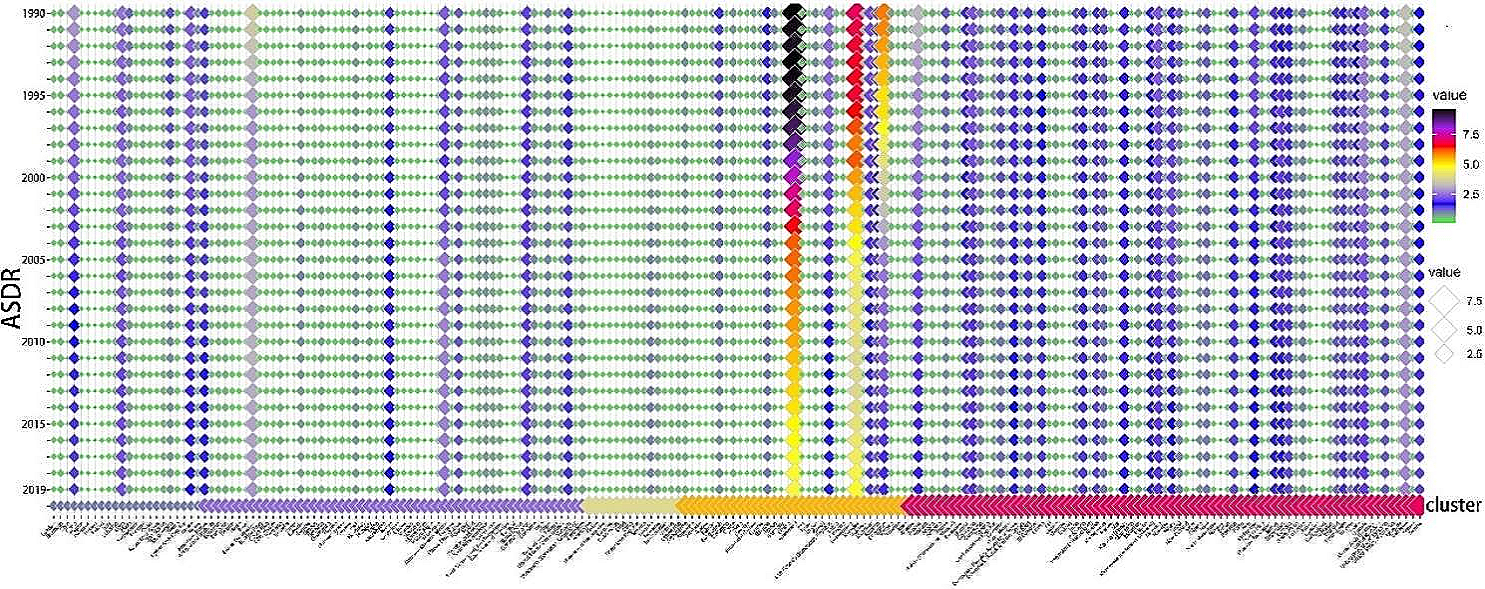
Alterations in ASDR of NPC in 200 countries or regions over the past 30 years

In terms of ASDR, there is a prevalent global downward trend (global EAPC-ASDR: -1.48 per 100,000), with about two-thirds of the countries or regions having an ASDR < 1.0 (per 100,000) and maintaining this incidence rate for a relatively long time (Fig. 5). It has been found that some countries or regions still maintain a high ASDR > 1.0 (per 100,000). Moreover, it is noteworthy that Greenland, Malaysia, Brunei Darussalam, and Viet Nam have long occupied the top four positions in the annual rankings for 30 years, and Singapore has dropped from third place in 1990 to eleventh place in 2019.

**Fig. 6:**
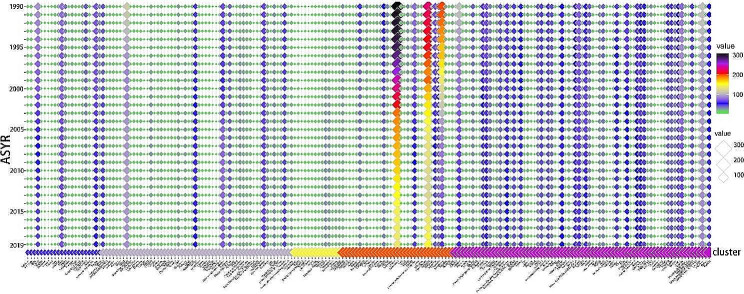
Alterations in ASYR of NPC in 200 countries or regions over the past 30 years

In terms of ASYR, there is a global downward trend observed (global EAPC-ASYR: -1.58 per 100,000), with about two-thirds of countries or regions having an ASYR < 20 (per 100,000), and very few countries displaying an ASYR > 100 (per 100,000) (Fig. 6). Malaysia, Greenland, Brunei Darussalam, and Viet Nam have long occupied the top four positions in the annual rankings for 30 years, whereas Singapore has dropped from third place in 1990 to tenth place in 2019.

### Group 3 could be the most at-risk subgroup

**Fig. 7:**
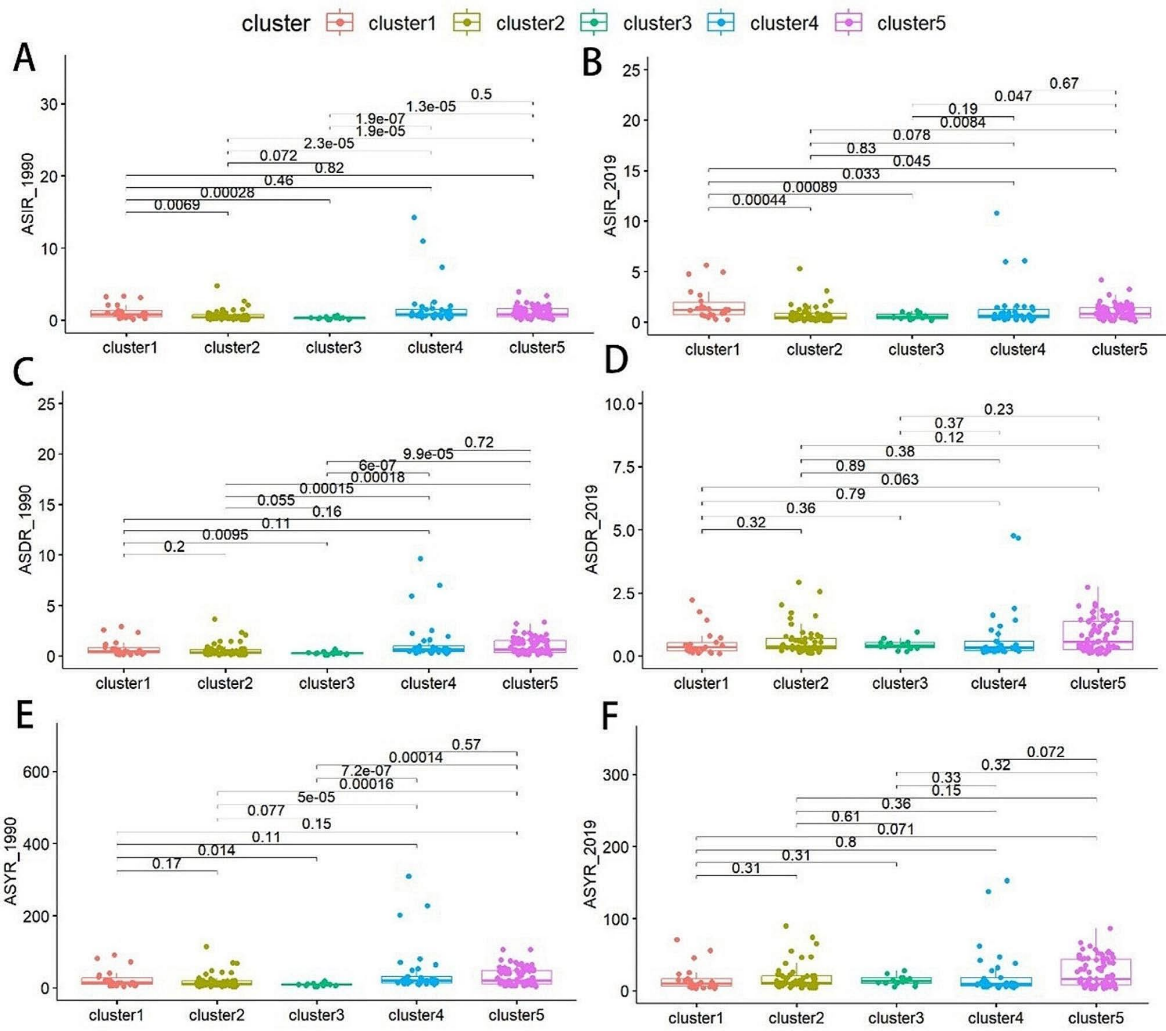
ASIR, ASDR and ASYR for 200 countries and regions in 1990 and 2019. **(A)** ASIR_1990; **(B)** ASIR_2019; **(C)** ASDR_1990; **(D)** ASDR_2019; **(E)** ASYR_1990; **(F)** ASYR_2019

After clustering the 200 different countries or regions into five distinct subgroups, we found that subgroup 3 was the most dangerous subgroup. For instance, in 1990, the ASIR, ASDR, and ASYR of subgroup 3 were significantly lower than those of subgroups 1, 4, and 5 (*p* < 0.05) (Fig. 7A, C and E), but higher than those of subgroup 2 (*p* > 0.05). However, over time, the ASIR, ASDR, and ASYR of subgroup 3 showed an increasing trend. Interestingly, by 2019, there was no statistically significant difference observed in ASIR between subgroup 3 and subgroups 2 and 4 (*p* > 0.05) (Fig. 7B), but there was a statistically significant difference noted between subgroup 3 and subgroup 1 and 5 (*p* < 0.05). In addition, in terms of ASDR, there was no statistically significant difference between the subgroup 3 and subgroups 1, 2, 4, and 5 (*p* > 0.05) (Fig. 7D). Similarly, in terms of ASYR, there was no statistically significant difference found between the subgroup 3 and the other subgroups (*p* > 0.05) (Fig. 7F), as was the case with ASDR.

## Discussion

This study makes full use of 30 years of NPC morbidity and mortality data, summarizes and groups the world, and clearly divides the world into 5 risk hierarchy: Group 1: The incidence rate showed an increasing trend whereas the mortality rate depicted a decreasing trend. Group 2: Morbidity as well as mortality showed a slight increase; Group 3: Morbidity as well as mortality increased significantly; Group 4: Morbidity and mortality decreased significantly; Group 5: Both morbidity as well as mortality decreased slightly. This risk stratification could aid to evaluate the effectiveness of health care policies and various strategies for NPC prevention as well as treatment in different countries or regions around the world.

Analyzing the most recent NPC incidence data, we found that NPC is still a local disease. NPC is a malignant tumor with high incidence reported in a few countries or regions, such as Singapore, Greenland, Malaysia, Brunei Darussalam, China, Tunisia, Libya, Algeria, Viet Nam, the Northern and Mariana Islands have occupied the top 10 positions in the ASIR rankings each year between 1990 and 2019. Moreover, in terms of the number of cases, Asia, and Southeast Asia still remain the hardest hit areas, including China, India, Japan, Vietnam, Malaysia, and other countries. This observation suggests that for countries with large populations, even with a small age-standardized incidence, there could be significantly higher number of cases. However, due to the large number of cases, even countries with rising ASIR, such as China, are more likely to captivate the research interest of scholars, attract the attention of the local governments, and promote adoption of more detailed tumor screening technology, thus forming scale effects. This can potentially lead to improvement in the treatment level, of patients, reduction in the mortality rate as well as the treatment cost, and effective mitigation of the social burden. This phenomenon may explain the reason behind increase in group 1 morbidity but decrease in mortality. Thus, for most countries or regions that have maintained a low incidence rate for 30 years, ASIR < 1, which is likely to be close to the natural incidence rate of NPC after excluding the high causative factors. This lower incidence could be primarily attributed to the various genetic differences, with environment and diet becoming secondary factors. This special phenomenon of ASIR polarization has attracted the attention of many renowned scholars who have conducted a lot of research in this area, but it is disappointing that the breakthrough is still not received.

According to the various previously published studies, the incidence of NPC may be closely related to EBV. Of course, several studies have also found that different environmental factors also play a part, such as passive smoking and household air pollution [16], which could be related to diet, such as the consumption of salted fish, salted vegetables, etc. Interestingly, some prior studies have also highlighted that oral health [17] is related to oral microbiota. Hence, reducing these controllable risk factors can markedly attenuate the incidence of NPC and the incidence of Group 4 has decreased significantly in 30 years, which is a good example. We noted that Malaysia was grouped into Group 4 in 2022 and a previous study has reported that Malaysia has a smoking prevalence of 22.8% [18], with exposure to second-hand smoke 21% indoors and 62% in public places. It has been observed that the Western Pacific region in which Malaysia is located has the highest percentage of smokers, according to WHO data [19]. Hence, in response to the social problem of high smoking rates, Malaysia has implemented several anti-smoking measures to reduce the tobacco abuse as well as second-hand smoke and the prevalence of smoking. As a consequence, the prevalence of current smoking and the susceptibility to smoking have all decreased significantly between 2003 and 2016. The number of people exposed to the second-hand smoking (SHS) in public places and at home has also decreased [20]. The decline in smoking rates could be one of the potential reasons for the rapid decline in NPC incidence in Malaysia. The infection rate of EBV virus remains high in the world [21]. However, there is no published literature available to report the specific change of EBV infection rate in countries with significant decrease of NPC incidence.

We found that ASIR, ASDR and ASYR were increasing in Group 3 countries or regions, and NPC control in Group 3 was relatively poor in comparison with other countries or regions in the world, thereby showing a dangerous signal. In the context of the global decline in morbidity and mortality, the rapid rise of ASIR, ASDR and ASYR in group 3 is particularly striking. Fortunately, the ASDR and ASIR of Group 3 have remained basically < 1 (per 100,000) for 30 years. However, increase at an alarming rate has been found in Group 3: ASIR (36.3-216.4%), ASDR (29.4-123.9%), ASYR (21.1-118.1%), thereby indicating that these countries or regions exhibit limited capacity in managing the pathogenic factors of NPC, and their proficiency early screening, diagnosis, and treatment of NPC is significantly deficient. we speculate that one of the reasons could be that the incidence of NPC is too small in these countries and belongs to the category of rare malignant tumors, hence there is little economic investment and energy in this field. However, exact reasons for this observation need more detailed research. We hope that this study can attract the attention of these countries or regions to the rapidly rising rate of NPC.

Globally, the mortality rate associated with NPC is decreasing, which is a positive indication of progress in the field of NPC pathophysiology and treatment. This is an encouraging sign for all oncology researchers. NPC is now considered to be a malignant tumor sensitive to both chemotherapy and radiotherapy (RT) after considerable development of science and technology. RT constitutes the main treatment for non-metastatic NPC [22]. In recent years, radiotherapy techniques have evolved greatly from traditional two-dimensional radiotherapy to three-dimensional conformal radiotherapy (3D-CRT), to more advanced intensive-modulated radiotherapy (IMRT) and stereotactic body radiotherapy (SBRT). It has been more than 90% of NPC patients who received high-quality IMRT after surgery and were well locally controlled [23]. It has to be mentioned that immunotherapy has further improved the therapeutic effect of NPC, for example, combination of PD-1/PD-L1 inhibitors with antiangiogenic inhibitor with molecular targeted agents, cancer vaccines, adaptive immunotherapy, and new ICI agents beyond PD-1/PD-L1 inhibitors in R/M NPC [24]. We look forward to more research in the future to improve the efficacy of NPC treatment.

In view of the rising incidence of NPC in the world, identification of effective strategies to reduce the incidence of NPC has become a major problem for the medical workers. Among the many possible pathogenic factors, EBV virus is currently considered to be closely correlated with risk of NPC. According to current research, there is a strong association between the Epstein-Barr virus (EBV) and the development of numerous malignant tumors [25], particularly NPC than be detected in 100% of non-keratinizing nasopharyngeal carcinomas [26]. Therefore, EBV-based tumor screening could be useful to facilitate early NPC diagnosis, improve treatment efficacy and reduce mortality. In a study involving 20,174 asymptomatic individuals it was found that individuals screened using real-time quantitative PCR to detect the plasma circulating EBV DNA in NPC patients, were more likely to have stage I-II disease at diagnosis (71% vs. 20%) and 3-year progression-free survival (PFS; 97% vs. 70%, *P* < 0.001) [27]. Moreover, in the screened population, plasma EBV DNA and serology were used, with positive predictive values of 11.1% and 4.8%, respectively [28, 29]. As for the cost, in a cost-benefit analysis published previously in 2020, it was reported that estimate the cost of plasma EBV DNA and EBV serology tests was $20.36 and $16.50, respectively [30]. Encouragingly, follow-up data from the next-generation screening methods based on EBV DNA fragment size and methylation pattern analysis suggested significantly improved EBV specificity, with positive predictive values of 19.6% and 35.1%, respectively [31]. In clinical practice, the selection of diagnostic tests for NPC typically follows a non-invasive to invasive approach as well as a cost-effective approach starting with cheaper options. As such, plasma EBV DNA and EBV serological tests are often utilized for NPC diagnosis due to their affordability. Despite their limitations in sensitivity and specificity, research suggests that new techniques may be developed in the future to enhance the accuracy of NPC diagnosis. These advancements could contribute to improved early screening and diagnosis of NPC, ultimately reducing the social burden associated with this disease.

## Conclusion

This is the first study to use EAPC and cluster methods to explore the potential NPC risk in 200 different countries or regions, and we observed that countries or regions in Group 3 were at the most at risk, with significant increases in ASIR, ASDR and ASYR.

## Data Availability

The datasets generated and/or analyzed during the current study are available in the GBD Data Tool repository (http://ghdx.healthdata.org/gbd-results-tool). This public link to GBD database is open, and the use of data does not require additional consent from IHME.
